# Single Cone Obturation versus Cold Lateral Compaction Techniques with Bioceramic and Resin Sealers: Quality of Obturation and Push-Out Bond Strength

**DOI:** 10.1155/2023/3427151

**Published:** 2023-01-17

**Authors:** Ahmad Nouroloyouni, Vahid Samadi, Amin Salem Milani, Sara Noorolouny, Haleh Valizadeh-Haghi

**Affiliations:** ^1^Department of Endodontics, School of Dentistry, Ardabil University of Medical Sciences, Ardabil, Iran; ^2^Department of Endodontics, School of Dentistry, Tabriz University of Medical Sciences, Tabriz, Iran; ^3^Department of Pediatric Dentistry, School of Dentistry, Ardabil University of Medical Sciences, Ardabil, Iran; ^4^Department of Operative Dentistry, School of Dentistry, Ardabil University of Medical Sciences, Ardabil, Iran

## Abstract

**Objectives:**

This study compared the obturation quality and push-out bond strength of single cone obturation (SCO) and cold lateral compaction (CLC) with AH-Plus and Sure Seal Root (SSR).

**Materials and Methods:**

This in vitro experimental study was conducted on 88 single-rootedsingle-canal teeth with straight roots that were randomly divided into four groups (*n* = 22). All teeth were decoronated and underwent cleaning and shaping. Obturation was performed with AH-Plus and SCO technique in group 1 (SAH), AH-Plus and CLC technique in group 2 (LAH), SSR and SCO technique in group 3 (SS), and SSR and CLC technique in group 4 (LS). The roots were then sectioned into 3-mm thick slices and underwent digital photography at x25 magnification to assess the quality of obturation in the coronal, middle, and apical thirds by Image J software. The PBS was measured by a universal testing machine. The mode of failure was also determined under a stereomicroscope.

**Results:**

The PBS was significantly higher in the LSS group than LAH and SAH groups, and also in the SSS group than the SAH group in all sections. The PBS in the LSS group was significantly higher than SSS in the coronal and middle thirds. Voids were significantly lower in LAH than in the SAH group in all sections. In LSS, voids in the coronal third were significantly lower than in LAH. In the middle third, voids in SSS were significantly lower than in SAH. The groups had no significant difference in the mode of failure (*P* > 0.05). The mean percentage of gutta-percha in the use of AH-Plus sealer was significantly higher than SSR (*P* < 0.05). The mean percentage of gutta-percha in the coronal third was lower than that in the middle and apical thirds (*P* < 0.05).

**Conclusion:**

SSR showed higher PBS and less voids than AH-Plus. High PBS of the CLC/SSR group showed that CLC should still be preferred to SCO, and in the case of using SCO, SSR should be preferred to AH-Plus.

## 1. Introduction

Root canal therapy is performed to resolve the root canal infection and prevent/eliminate periapical infection [1–3]. Three-dimensional sealing of debrided root canals is a critical step to prevent the reentry of microorganisms and their toxins into the root canal system and their extrusion into the periapical tissue, which can lead to treatment failure [3–5]. Endodontic sealers are essential for sealing the entire root canal length, apical foramen, root canal irregularities, and the gap between the root canal wall and the core root filling material [6]. According to Chandra [7], an ideal sealer should have properties such as excellent sealing ability after setting, adequate dimensional stability, optimal adhesion to the canal walls, and favorable biocompatibility [7]. At present, different types of sealers are available in the market including glass ionomer-based, zinc-oxide, and resin sealers. However, there is still a need for a sealer with more favorable properties [8]. Accordingly, bioceramic sealers were recently introduced [9].

Calcium silicate-based sealers are presented in the following two forms: (I) one-component sealers (ready-to-use), which are available in a premixed syringe with calibrated intracanal tips and utilize external water supply to set, and (II) two-component sealers with internal water supply [10].

Recently, a premixed injectable calcium silicate-based sealer known as Sure Seal Root (SSR) BC Sealer was introduced into the market. As stated by the manufacturer, it utilizes moisture to initiate and complete its setting reactions. After setting, a chemical bonding occurs with a void-free interface between the gutta-percha, sealer, and radicular dentin. The physicochemical properties of this sealer have been the topic of considerable attention. This sealer has an alkaline pH, optimal chemical stability, and high biocompatibility [12–14].

Different obturation techniques are available including the use of thermoplastic gutta-percha, cold lateral compaction (CLC), vertical condensation, and single-cone obturation (SCO) techniques. The CLC technique has a high level of safety, is cost-effective, and has shown favorable clinical results. It is the standard root canal obturation technique [3]. However, it has drawbacks such as a high level of difficulty, risk of void formation, and risk of vertical root fracture due to the application of wedging forces by instruments such as spreaders. Also, the CLC technique may have a suboptimal outcome due to incomplete obturation of curved canals. In the CLC technique, the correct use of a spreader may help in gaining more space for the insertion of accessory gutta-percha points [15].

The SCO technique is a subtype of the CLC technique in which one gutta-percha point is prepared with a taper compatible with the final shape and taper of the canal and is inserted into the canal, allowing complete obturation without any accessory points [15]. The SCO technique is often associated with a good outcome in the round, narrow, and regular root canals. However, the outcome may not be satisfactory in root canals with irregular shapes. This technique does not require compaction and is popular due to its simplicity and fast process. This technique requires a higher amount of sealer than the compaction and condensation techniques; thus, its outcome depends more on the properties of the sealer [15].

Optimal adhesion and adaptation of root-filling material to the canal walls play a fundamental role in the provision of the expected hermetic seal in endodontic treatment. Therefore, the push-out test is commonly used to quantitatively assess this adhesion [16].

This study aimed to compare the quality of root canal obturation and the push-out bond strength (PBS) of SCO and CLC techniques by using AH-Plus resin sealer and SSR bioceramic sealer.

## 2. Materials and Methods

This in vitro experimental study was conducted on 88 single-rooted, single-canal teeth with straight root canals as confirmed on two periapical radiographs taken at two different angles perpendicular to each other. The teeth had been extracted for purposes not related to this study (such as poor periodontal prognosis or orthodontic treatment). The study was approved by the ethics committee of Ardabil University of Medical Sciences (IR.ARUMS.REC.1398.212).

The teeth were stored in 0.05% sodium chloramine solution at room temperature since this solution is commonly used for disinfection and storage of extracted teeth in vitro [17, 18].

The inclusion criteria were (I) root length of 10 to 15 mm, (II) absence of severe curvature in the roots, absence of oval-shaped orifice, and absence of internal/external root resorption, and (III) initial file size not larger than #20.

The sample size was calculated to be 22 in each group (a total of 88 in 4 groups) according to a study by Krug et al. [19], assuming alpha = 0.05, beta = 0.2, and study power of 80%.

The teeth were inspected at x20 magnification and those with root cracks were excluded. The teeth were decoronated 1 mm above their cementoenamel junction (CEJ) by a diamond disc at low speed (Isomet, Buchler, Lake Bluff, USA) under water spray. At this step, teeth that did not meet the eligibility criteria were excluded and replaced. The working length was determined by using the largest file that reached the apex (Mani, Tochigi, Japan); 1 mm was subtracted from its length to determine the working length. At this step, teeth with an initial file size >20 were excluded and replaced. The teeth were then instrumented by using the gold-standard ProTaper rotary system according to the standard protocol of the manufacturer up to #F5. After using each file, the root canals were rinsed with 5.25% sodium hypochlorite with a side-vented needle. Next, the root canals were rinsed with 2 mL of 17% EDTA, followed by 2 mL of 5.25% sodium hypochlorite, and finally, with 4 mL of saline. The root canals were dried with paper points (PT Dent, USA) and were randomly divided into four groups (*n* = 22). The teeth were excluded and replaced in case of the occurrence of any procedural error.

### 2.1. Group 1 (SS)

A #F5 gutta-percha point was inserted into the canal to the working length. SSR (Sure Dent Corp., Gyeonggi-do, Korea) was then delivered into the coronal third of the root canal using an intracanal tip. Also, the apical third of gutta-percha was dipped in the sealer and gently inserted into the canal. Excess gutta-percha was cut at the CEJ, and the coronal part of gutta-percha was condensed with gentle pressure using an endodontic plugger.

### 2.2. Group 2 (SAH)

A #F5 gutta-percha was inserted into the canal to the working length. AH-Plus sealer (Dentsply DE Trey, Konstanz, Germany) was then delivered into the coronal third of the root canal using an intracanal tip. Also, the apical third of gutta-percha was dipped in the sealer and gently inserted into the canal. Excess gutta-percha was cut at the CEJ, and the coronal part of gutta-percha was condensed with gentle pressure using an endodontic plugger.

### 2.3. Group 3 (LS)

A #50 gutta-percha with 0.02 taper was inserted into the canal to the working length. Sure Seal Root was then delivered into the coronal third of the root canal using an intracanal tip. Also, the apical third of gutta-percha was dipped in the sealer and gently inserted into the canal. Next, a #30 spreader (Mani, Tochigi, Japan) was inserted into the canal adjacent to the master cone at a 0–2 mm distance from the working length. Accessory gutta-percha points (#25/0.02) were placed in the space created by the spreader immediately after its removal. Excess gutta-percha was cut at the CEJ, and the coronal part of gutta-percha was condensed with gentle pressure using an endodontic plugger.

### 2.4. Group 4 (LAH)

A #50 gutta-percha with 0.02 taper was inserted into the canal to the working length. AH-Plus was then delivered into the coronal third of the root canal using an intracanal tip. Also, the apical third of gutta-percha was dipped in the sealer and gently inserted into the canal. Next, a #30 spreader (Mani, Tochigi, Japan) was inserted into the canal adjacent to the master cone at a 0–2 mm distance from the working length. Accessory gutta-percha points (#25/0.02) were placed in the space created by the spreader immediately after its removal. Excess gutta-percha was cut at the CEJ, and the coronal part of gutta-percha was condensed with gentle pressure using an endodontic plugger.

All phases of cleaning and obturation of root canals were performed by the same operator. A radiograph was then obtained from all specimens to ensure the optimal quality of root canal filling. Teeth with voids or other detectable procedural errors on radiographs were excluded and replaced. The teeth were then incubated at 37°C and 100% humidity for 14 days to allow the complete setting of sealers.

### 2.5. Quality of Obturation with Gutta-Percha and Sealer

The teeth were fixed with cyanoacrylate glue and horizontally sectioned in the apical, middle, and coronal thirds to obtain slices with a maximum thickness of 3 mm [20]. A low-speed diamond disc (Isomet, Buchler, Lake Buff, USA) was used for this purpose under water spray. The slices were then inspected under a stereomicroscope (Expert DN) at x25 magnification and were digitally photographed. The teeth with oval-shaped root canals or isthmuses were excluded and replaced. The areas were filled with gutta-percha and sealer, and the voids were quantified by Image J software (National Institutes of Health, public domain) and reported as a percentage. This process was repeated by another operator, and the obtained values were recorded. The level of agreement between the two observations was found to be 1, indicating excellent agreement.

### 2.6. PBS Test

Each specimen was subjected to apico-coronal force application along the longitudinal axis of the tooth. The load was applied by cylindrical rods with 0.45, 0.6, and 0.9 mm diameters in a universal testing machine (Hounsfield Test Equipment, model: H5K-S, England). For each specimen, a rod that covered approximately 90% of the surface of root-filling material was selected. The load was applied at a crosshead speed of 0.5 mm/minute until fracture. The maximum load at fracture was recorded in Newtons (N). The PBS in Newtons was converted to megapascals (MPa) by dividing the load (N) by the cross-sectional area of the entire surface subjected to load application, which was calculated using the following formula:

(1) $$\begin{array}{c} area:\pi \left[\frac{a1∙a2}{2}\right]∙h\text{,} \end{array}$$

where *a*1 is the canal diameter in the coronal part of the slice, *a*2 is the canal diameter in the apical part of the slice, and *h* is the slice thickness. The specimens were then inspected at x20 magnification to determine the mode of failure, which was categorized as cohesive (fracture within the filling material), adhesive (fracture at the interface of dentinal wall and filling material), and mixed (a combination of adhesive and cohesive).

### 2.7. Statistical Analysis

Data were analyzed using SPSS version 22. The Kolmogorov–Smirnov test was used to analyze the normality of data distribution. An independent *t*-test was used to compare the percentage of canals filled with a sealer, and the Mann–Whitney *U* test was used to compare the PBS and voids among the groups. Inter-grouptwo-way and three-way ANOVA were applied to compare the percentage of canals filled with gutta-percha, and the Chi-square test was used to analyze the correlation between the failure mode and the study group. The level of significance was set at 0.05.

## 3. Results

Table 1 presents the mean and standard deviation of PBS, percentage of gutta-percha, percentage of voids, and percentage of sealer in the study groups.

### 3.1. PBS

The Kolmogorov–Smirnov test showed the non-normal distribution of PBS data (*P* < 0.001). Thus, the Kruskal–Wallis test was applied to compare the PBS of the study groups, which showed a significant difference (*P* < 0.001). Pairwise comparisons by the Mann–Whitney *U* test (Table 2) showed that the mean PBS in the LSS group was significantly higher than LAH and SAH groups in the coronal, middle, and apical thirds. The mean PBS in the SSS group was significantly higher than the SAH group in the coronal, middle, and apical thirds (*P* < 0.05). The mean PBS in the SSS group was significantly higher than the SAH group (*P* < 0.05). The mean PBS in the LSS group was significantly higher than the SSS group in the coronal and middle thirds (*P* < 0.05). The mean PBS in the SSS group was significantly higher than the LAH group in the middle and apical thirds (*P* < 0.05). No other significant differences were noted.

### 3.2. Percentage of Voids

The Kolmogorov–Smirnov test showed a non-normal distribution of the percentage of voids (*P* < 0.001). Thus, the groups were compared in this regard by the Kruskal–Wallis test, which showed no significant difference among the study groups in the percentage of voids (*P* > 0.05).

### 3.3. Percentage of Gutta-Percha

The Kolmogorov–Smirnov test showed normal distribution of the percentage of gutta-percha data (*P* > 0.05). Thus, three-way ANOVA was applied to compare the groups in this regard and assess the effect of the technique of obturation, type of sealer, and area of the root (coronal, middle, or apical) on the percentage of gutta-percha. The results showed no significant difference in the mean percentage of gutta-percha between the two obturation techniques (*P* > 0.05). A significant difference existed in the mean percentage of gutta-percha between the two sealers, and the mean percentage of gutta-percha in AH Plus was significantly higher than SSR (*P* < 0.001). Also, a significant difference existed in the mean percentage of gutta-percha among the coronal, middle, and apical thirds (*P* < 0.05). Pairwise comparisons showed that the mean percentage of gutta-percha in the coronal third was significantly lower than in the middle and apical thirds (*P* < 0.05). No other significant differences were found in this regard (*P* > 0.05).

Independent samples *t*-test was used to analyze the effect of the obturation technique on the mean percentage of gutta-percha based on the two sealer types. The results are presented in Table 3.

### 3.4. Mean Sealer Percentage

Table 4 presents the mean sealer percentage in the study groups. Three-way ANOVA was applied to analyze the effect of the obturation technique on the mean sealer percentage based on the type of sealer and the area of the root (coronal, middle, and apical third). The results showed significant effects of type of sealer (*P* < 0.001), area of the root (*P* < 0.001), interaction of obturation technique and sealer type (*P* = 0.001), and the interaction of obturation technique, sealer type, and area of the root (*P* = 0.042) on the mean sealer percentage. Thus, pairwise comparisons of the root areas were carried out by the Games–Howell test and the results are reported in Table 5.

### 3.5. Mode of Failure

Group and mode of failure were not significantly correlated, and the frequency of modes of failure was not significantly different among the groups (*P* > 0.05).

## 4. Discussion

A hermetic apical seal is imperative for a successful endodontic treatment to prevent the leakage of fluids and materials from the periradicular tissue into the root canal system and vice versa [21, 22]. According to Ramezanali et al. [23], many endodontic problems are due to incomplete root canal sealing. Gutta-percha, in combination with sealer, serves as the gold standard for root canal obturation due to optimal biocompatibility, no toxicity or allergic reactions, and easy retrieval from the root canal system. However, it has shortcomings such as the inability to increase the root strength since it cannot bond to dentin and the incomplete filling of the root canal space [24]. Evidence shows that PBS cannot directly predict the clinical success of treatments. However, it provides valuable information regarding the comparison of sealers and different obturation techniques [3, 25]. Thus, the PBS was measured in this study to assess the bond strength of root-filling materials to root canal walls and the efficacy of the CLC obturation technique. The CLC technique was selected in this study since it is currently the most commonly used obturation technique [3, 25]. The SCO technique is also suggested by the manufacturers of the new generation of bioceramic sealers [3, 25]. The mean PBS in the LSS group was significantly higher than LAH and SAH groups in the coronal, middle, and apical thirds. The mean PBS in the SSS group was significantly higher than the SAH group in the coronal, middle, and apical thirds (*P* < 0.05). The mean PBS in the SSS group was significantly higher than the SAH group (*P* < 0.05). The mean PBS in the LSS group was significantly higher than the SSS group in the coronal and middle thirds (*P* < 0.05). The mean PBS in the SSS group was significantly higher than the LAH group in the middle and apical thirds (*P* < 0.05). In line with the present results, O'Brien et al. [18] 2020 compared the PBS of AH-Plus and CeraSeal bioceramic sealer and reported that the mean PBS in the bioceramic sealer group was higher than that in the AH-Plus group. To explain the results, it should be stated that Sure Seal Root bioceramic sealer forms chemical bonds to dentin through the synthesis of hydroxyapatite during its setting reactions, and thus, higher bond strength is achieved in the use of this sealer. Also, Sure Seal Root is easily infused into the dentinal tubules and creates a hermetic seal [16].

The percentage of root canal filling was also evaluated in this study. Gutta-percha is the most commonly used root-filling material. Sealers are used for better adaptation of gutta-percha to the root canal walls. Since the dimensional stability of gutta-percha is higher than that of sealer, the percentage of gutta-percha should be as high as possible, and the percentage of sealer should be lower in an ideal obturation [26–28]. Accordingly, the present study quantified the percentage of gutta-percha, sealer, and voids in different sections. The current results showed no significant difference in the mean percentage of gutta-percha between the two obturation techniques, which is similar to the past studies [29–31].

In the present study, the mean percentage of sealer in the AH-Plus group was significantly lower than SSR (*P* < 0.001). This finding can be due to the fact that bioceramic sealers undergo a little expansion rather than shrinkage [32]. Also, the mean percentage of gutta-percha was significantly different among the coronal, middle, and apical thirds, and it was lower in the coronal third than the middle and apical thirds. The discrepancy between the canal shape and file shape increases in the coronal third due to greater root canal irregularities in this area, which can be one reason for the higher percentage of residual sealer in the coronal third [33]. Finally, based on the present results, the percentage of voids did not differ significantly among the groups. Both obturation techniques and also both sealers produced voids, which is in accordance with the findings of Celikten et al. [34], who suggested that voids are mainly correlated with the root canal anatomy rather than the root canal filling material or technique.

## 5. Strengths

This study compared two common obturation techniques qualitatively and quantitatively.

## 6. Limitations

This study had an in vitro design. Thus, a complete simulation of the clinical setting was not possible. Also, when sectioning the teeth, smearing of the filling material on the sectioned surface may occur despite water cooling. Unnoticed smearing might have influenced the accurate measurement of small voids. Moreover, when using sliced sections, only a 2-dimensional assessment of void areas can be performed. Thus, to overcome these shortcomings, additional use of nondestructive scans of the teeth would be beneficial.

## 7. Conclusion

Sure Seal Root has better physical, chemical, and sealing properties compared to AH-Plus. The high push-out bond strength of lateral compaction/Sure Seal group showed that cold lateral compaction should still be preferred to single-cone obturation. In the case of using the single cone technique, SSR is superior to AH Plus.

## Figures and Tables

**Table 1:** Mean and standard deviation of PBS, percentage of gutta-percha, percentage of voids, and percentage of sealer in the study groups.

Group	Push-out bond strength		Gutta-percha percentage		Void percentage		Sealer percentage	
	Mean	Std. deviation	Mean	Std. deviation	Mean	Std. deviation	Mean	Std. deviation
LAHC	1.308	0.466	61.8288	13.35621	0.69361464	0.859791292	37.4776	13.38830
LAHM	1.199	0.395	69.2478	10.50286	0.89518280	1.063389173	29.8961	10.27965
LAHA	1.388	0.658	64.1757	12.99882	1.09696667	1.727413163	33.3160	10.59724
LSC	1.993	0.969	56.8768	12.38811	1.68737949	2.044482213	41.4358	12.29125
LSM	2.827	1.357	67.9241	13.66464	1.66681164	1.809702236	30.4091	12.74406
LSA	3.102	1.140	60.7299	10.69044	1.98173853	1.826335429	37.2883	11.11168
SAHC	1.091	0.460	58.3002	6.86907	1.38225485	1.003168116	40.2506	6.71463
SAHM	1.087	0.534	78.2242	12.69152	2.50237359	1.854858469	19.2734	12.33088
SAHA	1.551	0.518	76.3376	8.05079	2.78554508	2.157673344	20.9681	6.64868
SSC	1.455	0.500	55.3457	16.56989	1.26726504	1.378866704	43.3870	16.44061
SSM	1.639	0.766	63.1666	11.91185	1.28851810	1.228987673	35.5449	11.76985
SSA	2.614	1.016	59.1645	13.49402	1.77899073	1.937642616	39.0565	13.14884

LAHC: lateral compaction, AH-plus, coronal section; LAHM: lateral compaction, AH-plus, middle section; LAHA: lateral compaction, AH-plus, apical section; LSC: lateral compaction, sure seal root, coronal section; LSM: lateral compaction, sure seal root, middle section; LSA: lateral compaction, sure seal root, apical section; SAHC: single cone, AH-plus, coronal section; SAHM: single cone, AH-plus, middle section; SAHA: single cone, AH-plus, apical section; SSC: single cone, sure seal root, coronal section; SSM: single cone, sure seal root, middle section; SSA: single cone, sure seal root, apical section.

**Table 2:** Comparison of PBS of the groups (Mann–Whitney U).

Paired groups compared	Mean rank	*P* value
LAHC	23.39	0.647
LAHM	21.61	

LAHC	21.82	0.725
LAHA	23.18	

LAHC	17.80	0.015^∗^
LSC	27.20	

LAHC	14.68	<0.001^∗^
LSM	30.32	

LAHC	12.18	<0.001^∗^
LSA	32.82	

LAHC	25.48	0.124
SAHC	19.52	

LAHC	25.43	0.130
SAHM	19.57	

LAHC	19.55	0.127
SAHA	25.45	

LAHC	20.68	0.348
SSC	24.32	

LAHC	19.16	0.084
SSM	25.84	

LAHC	14.02	<0.001^∗^
SSA	30.98	

LAHM	21.27	0.526
LAHA	23.73	

LAHM	17.00	0.005^∗^
LSC	28.00	

LAHM	14.23	<0.001∗
LSM	30.77	

LAHM	11.73	<0.001^∗^
LSA	33.27	

LAHM	24.00	0.439
SAHC	21.00	

LAHM	25.11	0.177
SAHM	19.89	

LAHM	18.27	0.029^∗^
SAHA	26.73	

LAHM	18.50	0.039^∗^
SSC	26.50	

LAHM	18.05	0.021^∗^
SSM	26.95	

LAHM	13.84	<0.001^∗^
SSA	31.16	

LAHA	18.98	0.069
LSC	26.02	

LAHA	15.36	<0.001^∗^
LSM	29.64	

LAHA	13.32	<0.001^∗^
LSA	31.68	

LAHA	25.55	0.116
SAHC	19.45	

LAHA	25.48	0.124
SAHM	19.52	

LAHA	20.75	0.366
SAHA	24.25	

LAHA	21.55	0.622
SSC	23.45	

LAHA	20.66	0.342
SSM	24.34	

LAHA	14.86	<0.001^∗^
SSA	30.14	

LSC	18.43	0.036^∗^
LSM	26.57	

LSC	16.34	0.001^∗^
LSA	28.66	

LSC	29.27	<0.001^∗^
SAHC	15.73	

LSC	28.77	0.001^∗^
SAHM	16.23	

LSC	25.45	0.127
SAHA	19.55	

LSC	26.77	0.027^∗^
SSC	18.23	

LSC	24.27	0.360
SSM	20.73	

LSC	18.41	0.035^∗^
SMA	26.59	

LSM	20.57	0.318
LSA	24.43	

LSM	31.32	<0.001^∗^
SAHC	13.68	

LSM	31.18	<0.001^∗^
sahm	13.82	

LSM	28.95	0.001^∗^
SAHA	16.05	

LSM	29.59	<0.001^∗^
SSC	15.41	

LSM	28.18	0.003^∗^
SSM	16.82	

LSM	22.77	0.888
SSA	22.23	

LSA	33.23	<0.001^∗^
SAHC	11.77	

LSA	32.82	<0.001^∗^
SAHM	12.18	

LSA	31.86	<0.001^∗^
SAHA	13.14	

LSA	32.64	<0.001^∗^
SSC	12.36	

LSA	30.55	<0.001^∗^
SSM	14.45	

LSA	24.27	0.360
SSA	20.73	

SAHC	22.80	0.879
SAHM	22.20	

SAHC	17.16	0.006^∗^
SAHA	27.84	

SAHC	18.39	0.034^∗^
SSC	26.61	

SAHC	17.55	0.011^∗^
SSM	27.45	

SAHC	13.59	<0.001^∗^
SSA	31.41	

SAHM	17.11	0.005^∗^
SAHA	27.89	
SAHM	17.68	0.013^∗^
SSC	27.32	

SAHM	17.75	0.014^∗^
SSM	27.25	

SAHM	13.73	<0.001^∗^
SSA	31.27	

SAHA	23.36	0.656
SSC	21.64	

SAHA	20.98	0.432
SSM	24.02	

SAHA	15.23	<0.001^∗^
SSA	29.77	

SSC	19.55	0.127
SSM	25.45	

SSC	14.59	<0.001^∗^
SSA	30.41	

SSM	15.95	0.001^∗^
SSA	29.05	

^∗^Presence of a significant difference at *P* < 0.05

**Table 3:** Effect of obturation technique on the mean percentage of gutta-percha based on the two sealer types (independent samples *t*-test).

Sealer type	Obturation technique	Mean	Std. deviation	*P* value
AH plus	Cold lateral compaction	65.0200	12.57216	0.006
	Single cone	71.3494	12.99145	

Sure seal	Cold lateral compaction	61.8436	12.96584	0.272
	Single cone	59.2256	14.27333	

**Table 4:** Mean sealer percentage in the study groups.

Obturation technique	Sealer type	Coronal		Middle		Apical	
		Mean	Std. deviation	Mean	Std. deviation	Mean	Std. deviation
Lateral	AH plus	37.4776	13.38830	29.8961	10.27965	33.3160	10.59724
	Sure seal	41.4358	12.29125	30.4091	12.74406	37.2883	11.11168
	All	39.4567	12.85799	30.1586	11.47138	35.3484	10.92008

Single cone	AH plus	40.2506	6.71463	19.2734	12.33088	20.9681	6.64868
	Sure seal	43.3870	16.44061	35.5449	11.76985	39.0565	13.14884
	All	41.8935	12.72203	27.4092	14.47898	30.4430	13.87958

All	AH plus	38.7981	10.70830	24.4613	12.45839	27.2926	10.77942
	Sure seal	42.4114	14.37910	32.9770	12.39833	38.1724	12.06376
	All	40.6468	12.77523	28.7681	13.07662	32.9245	12.63877

**Table 5:** Pairwise comparisons of the root areas regarding the percentage of sealer by the Games–Howell test.

Group (I)	Group (J)	Mean difference (I-J)	*P* value
Coronal	Medial	11.8787	<0.001
	Apical	7.7222	<0.001

Medial	Apical	−4.1564	0.089

## Data Availability

The data used to support the findings of this study were supplied by the corresponding author under license and data will be available on request. Requests for access to these data should be made to the corresponding author.
